# Functional stability despite structural changes in freshwater biofilm communities exposed to an antibiotic and an herbicide - the role of nutrient conditions

**DOI:** 10.1093/femsec/fiaf094

**Published:** 2025-09-24

**Authors:** Sophie Oster, Eric Bollinger, Verena C Schreiner, Tobias Schmitt, Sabine Filker, Mirco Bundschuh

**Affiliations:** iES Landau, Institute for Environmental Sciences, University of Kaiserslautern-Landau (RPTU), Fortstraße 7, Landau 76829, Germany; iES Landau, Institute for Environmental Sciences, University of Kaiserslautern-Landau (RPTU), Fortstraße 7, Landau 76829, Germany; Faculty of Biology, University of Duisburg-Essen, Universitätsstraße 2, Essen 45141, Germany; Research Center One Health Ruhr, University Alliance Ruhr, Universitätstraße 2, Essen 45141, Germany; iES Landau, Institute for Environmental Sciences, University of Kaiserslautern-Landau (RPTU), Fortstraße 7, Landau 76829, Germany; Faculty of Biology, Department of Ecology, University of Kaiserslautern-Landau (RPTU), Erwin-Schrödinger-Straße 14, Kaiserslautern 67663, Germany; iES Landau, Institute for Environmental Sciences, University of Kaiserslautern-Landau (RPTU), Fortstraße 7, Landau 76829, Germany; Department of Aquatic Sciences and Assessment, Swedish University of Agricultural Sciences, Lennart Hjelms väg 9, Uppsala, SE-756 51, Sweden

**Keywords:** ciprofloxacin, functional redundancy, metabarcoding, periphyton, primary production, propyzamide

## Abstract

Freshwater autotrophic biofilms play a vital role in primary production and nutrient cycling in freshwater ecosystems but are increasingly exposed to chemical stressors such as antibiotics or herbicides. Although nutrient availability may modulate biofilm sensitivity, its impact on biofilm responses to these stressors remains poorly understood. In four independent experiments, we investigated the functional (ash-free dry weight and chlorophyll a, b and c) and structural (16S/18S rRNA metabarcoding) responses of stream-derived biofilms under low- and high-nutrient levels to chronic exposure (14 days) to the antibiotic ciprofloxacin and the herbicide propyzamide in laboratory stream microcosms. High-nutrient levels strongly increased biofilms functional responses and altered the community composition. Chemical exposure led to pronounced shifts in prokaryotic (ciprofloxacin) and eukaryotic (propyzamide) communities, but without significant effects on functional responses, suggesting functional redundancy and ecological buffering capacity of freshwater biofilms. These results highlight the critical role of nutrient supply in biofilm responses and the need for caution when extrapolating laboratory results to field conditions.

## Teaser

This study shows that freshwater biofilms maintain stable function despite structural shifts from chemical stress, highlighting their functional redundancy under varying nutrient conditions.

## Introduction

Autotrophic biofilms are crucial components of freshwater ecosystems (Wetzel 1983, Battin et al. 2016), substantially contributing to primary production and nutrient cycling (C, N and P). Additionally, they serve as a food source for primary consumers and, depending on the thickness, as a habitat. Biofilms are composed of algae, bacteria and fungi, embedded in an extracellular matrix (Wu 2017). In rivers, omnipresent chemical stressors such as pesticides (Tang et al. 2021) and pharmaceuticals (Wilkinson et al. 2022) can lead to severe changes in biofilm community structure and function, affecting food-webs bottom-up (Doose et al. 2021, Konschak et al. 2021). For several decades, a vast amount of research has investigated freshwater biofilm responses to various chemical stressors. Due to the high biodiversity within biofilms, chemicals may induce a wide range of effects, which depend on the chemicals’ respective mode of toxic action, but also the successional state of the biofilm. Herbicides, for example, may primarily affect photo-autotrophic organisms, including diatoms (Ahrens and Edwards 1994), while their impact may be most prominent when biofilms are dominated by photo-autotrophs in abundance (i.e. during spring and summer, Beck et al. 2019). In contrast, antibiotics are designed to primarily affect bacteria (Kohanski et al. 2010) and their impact may be more pronounced when the biofilm is dominated by heterotrophs (i.e. during autumn and winter).

Besides the properties of chemical stressors, other factors may determine the biofilms sensitivity and response, which is usually recorded in changes of functional (such as biomass, primary production, nutrient cycling) and structural parameters (i.e. community composition). The availability of nutrients can affect responses of both autotrophs (algae) and heterotrophs (bacteria; (Dodds & Smith,^2016^)). Specifically, nutrient enrichment often leads to an increase in autotrophic generalist biomass, altering biofilm community composition and potentially reducing the reliance on heterotrophic processes (Scott et al. 2008). Conversely, in low-nutrient environments, heterotrophic bacteria may dominate, playing a crucial role in nutrient recycling and biofilm maintenance. Thus, fluctuations in nutrient concentrations alter biofilm's functional and structural parameters, which can be reflected in its tolerance to chemical stressors (Navarro et al. 2002) as informed by the Dynamic Energy Budget (DEB) theory (Kooijman 2010).

To the best of our knowledge remain the effects of chemicals under different nutrient conditions on the structure and function of freshwater biofilms poorly understood. To address this knowledge gap, we conducted four independent experiments using artificial stream microcosms. We examined the responses of stream-derived but laboratory-colonised biofilms to chronic (14-day) exposure to two chemical stressors with different modes of action, each tested under two nutrient conditions (low vs. high). As chemical stressors, we used the antibiotic ciprofloxacin (a topoisomerase II/IV inhibitor) and the herbicide propyzamide (a microtubule assembly inhibitor). Ciprofloxacin experiments were conducted in winter and propyzamide experiments in summer, as an increased incidence of antibiotics in winter and herbicides in summer was assumed (Kim et al. 2023, Cao et al. 2024). Ciprofloxacin, a wide spectrum fluoroquinolone antibiotic, is frequently detected downstream of wastewater treatment plant effluents, often reported to alter microbial community structure and selecting for resistant strains even at sublethal levels (Kohanski et al. 2010, Danner et al. 2019). In contrast, propyzamide is a selective benzamide herbicide primarily preventing plant cell division (Lewis et al. 2016). It is widely applied in agriculture, with seasonal peaks in surface waters following application events (Liess et al. 2021). Biofilm responses were assessed on a functional basis using ash-free dry weight (AFDW) and chlorophyll a, b, and c concentrations, and on a structural basis via DNA metabarcoding of prokaryotic and eukaryotic communities. We hypothesised (i) the antibiotic ciprofloxacin to primarily affect the prokaryotic and the herbicide propyzamide the eukaryotic community composition. The whole biofilm community was hypothesised (ii) to harbour more generalists and a lower diversity if cultured under high- relative to low-nutrient conditions, ultimately affecting biofilm sensitivity—defined here as the extent to which functional and structural parameters respond to stress. Moreover, (iii) alterations in the community composition were expected to be more pronounced in response to nutrient levels relative to the applied chemicals, as nutrient availability more directly shapes biofilm structure, while chemical exposure effects are often subtler or stress dependent (Dodds & Smith, 2016). Finally, we anticipated (iv) a dose-dependent response of functional parameters, with nutrient enrichment increasing and chemical exposure decreasing biofilm AFDW and chlorophyll concentrations.

## Material and methods

### Experimental design

The four independent experiments were carried out in the Landau Laboratory Stream Microcosm facility of the RPTU Kaiserslautern-Landau (Landau, Germany). The microcosm facility was equipped with stainless-steel channels (120×30×20 cm, 40 L of artificial media), each containing a stainless-steel paddle wheel to simulate running water conditions at a flow velocity of 0.02 $ \pm $ 0.01 m s^−1^ (Fig. S1). The experimental design was consistent across all four experiments, with nutrient conditions (low vs. high) and chemical stressors (ciprofloxacin or propyzamide) systematically varied to assess their individual effects (Table 1; exact dates of the experiments can be found in the supplements Table S1). Biofilms were colonised and exposed under two nutrient conditions: the high-nutrient Kuhl medium (Kuhl and Lorenzen 1964), which is used to culture algae in the laboratory and was intended to simulate eutrophic water in our experiments and the rather low-nutrient modified SAM-S5 medium (Rybicki et al. 2012), which resembles mesotrophic water.

**Table 1.** Overview of the experiments.

Experiment	Season	Nutrients	Chemical Stressor
1	Winter	Low	Ciprofloxacin
2	Winter	High	Ciprofloxacin
3	Summer	Low	Propyzamide
4	Summer	High	Propyzamide

Season: Winter or Summer, Low-nutrient medium: modified SAM-S5 (mesotrophic conditions), High-nutrient medium: Kuhl (eutrophic conditions). Chemical Stressor: Ciprofloxacin or Propyzamide. The ingredients of both media can be found in the supplementary data (Table S2 and S3).

Biofilm sampling was carried out in the same way for the four independent experiments: Biofilm was sampled by collecting ten medium-sized, biofilm-covered stones (∼20 cm diameter) from the naturally low-nutrient Sulzbach river (∼70 µS cm^−1^ conductivity, <0.05 mg L^−1^ phosphate, ∼0.7 mg L^−1^ nitrate; Filter Photometer Nanocolor^®^ 500 D, Macherey-Nagel, Düren, Germany), located within a nature reserve (49°14′N; 7°57′E; ∼8 h sunlight per day). Therefore, new stones were collected from the same place for each experiment. To determine each initial biofilm community, a biofilm subsample was brushed off some stones on site, fixed in RNA preservation liquid (LifeGuard^®^, Qiagen, Hilden, Germany) and frozen at -20°C. For the experiments, biofilm-covered stones were transported to the laboratory immersed in river water, where the biofilm was scraped off stones using sterile toothbrushes and poured through a 50 µm sieve to remove leaf residues, macroinvertebrates and sediment. The filtrate was homogenised in 10 L filtered (50 µm) river water overnight on a magnetic stirrer at 200 r/m. The next day, aliquots of the filtrate were added to indoor microcosm channels and served as inoculum to colonise on white unglazed, sterile ceramic tiles (4.7×4.7 cm, 20 per channel). As four ciprofloxacin concentrations, including a control were tested in triplicates (0, 1, 10, 100, or 1000 µg L^−1^), 15 channels were used in the ciprofloxacin experiments (1 & 2, Table 1). In the propyzamide experiments (3 & 4), one additional concentration was tested (10,000 µg L^−1^), resulting in 18 used channels. Accordingly, 660 ml of inoculum was aliquoted in experiments 1 & 2 and 550 ml in experiments 3 & 4.

The initial and chemical-free colonisation phase varied in its duration between the four experiments (2–8 weeks, Table S1) due to the difference in nutrients, daylight regime (16:8 h light: darkness schedule for summer experiments, 10:14 h for winter experiments) and water temperature, which all affect growth directly, resulting in a faster biofilm development under high-nutrient conditions and in summer. The water temperature was monitored throughout the study and averaged 18±2°C in summer and 15±3°C in winter. Half of the medium (20 L) was changed weekly, compensating for evaporation and providing fresh nutrients. Chronic (14-day) chemical exposure was initiated once the biofilm reached a visibly mature stage, as indicated by structural development and consistent biomass accumulation (after a minimum of two weeks of growth). Channels were randomly spiked with nominal concentrations of ciprofloxacin hydrochloride hydrate (98% (Acros Organics, Geel, Belgium) or propyzamide (formulation Kerb^TM^ Flo, Dow AgroSciences, Indianapolis, United States). The concentration selection was based on environmentally realistic concentrations, as ciprofloxacin has been detected in low µg L^−1^ ranges downstream of wastewater effluents (Meffe and De Bustamante 2014, Danner et al. 2019) and propyzamide was found at a concentration of 0.58 µg L^−1^ in small streams (Liess et al. 2021). In addition, an ascending concentration series was selected to investigate the biofilm responses at higher concentrations. The two nutrient conditions differed in both composition and concentration (Table S2 & S3; low-nutrient modified SAM-S5, Rybicki et al. 2012: ∼400 µS cm^−1^ conductivity, <0.2 mg L^−1^ phosphate, ∼6 mg L^−1^ nitrate and high-nutrient Kuhl medium, Kuhl and Lorenzen 1964: ∼2000 µS cm^−1^ conductivity, >300 mg L^−1^ phosphate, 950 mg L^−1^ nitrate). Both media provided elevated nutrient levels relative to the source stream of the biofilm communities.

Sodium-vapor (400 W) and metal halide lamps (SON-T Agro and Master HPI-T Plus, Koninklijke Philips N.V., Amsterdam, Netherlands) generated an illuminance around 14 klux at a wavelength of 400–700 nm, supporting photosynthesis and biofilm growth. The channels were placed in a cooled water bath (Lauda VC 1200, Lauda Dr. R. Wobser GmbH & Co. KG, Lauda-Königshofen, Germany) to minimise temperature fluctuation in response to the energy emission of the lamps. At the end of the chemical exposure phase (Day 14), two tiles per endpoint were randomly sampled from each channel, biofilm was scraped off using sterile razor blades and stored at -20°C until further analyses.

### Functional parameters

To investigate functional biofilm responses, we analysed two biomass markers, AFDW and photosynthetic pigment concentrations (i.e. chlorophyll a, b and c). To determine the total organic content in form of AFDW, samples were defrosted and filtered through pre-ashed and pre-weighed glass fibre filters (GF 6 pore size 1 µm, Whatman, Dassel, Germany). Filters were subsequently dried for 24 h at 60°C, weighed to the nearest 0.01 mg, burned for 5 h at 500°C, and reweighed. AFDW was calculated as per Biggs and Kilroy (2000) and normalised to the area of two tiles (i.e. mg cm^−2^).

To extract photosynthetic pigments, biofilm samples were added to 5 ml 90% acetone and stored overnight at -20°C. The next day, samples were treated with ultrasound for 2 min and then centrifuged for 15 min at 4°C and 3500 r/m. The supernatant (300 µL) was transferred in triplicates to a transparent 96-microwell plate. Pigments were quantified via spectrophotometry using a wavelength spectrum of 450-750 nm (Microplate reader, Infinite 200 PRO, Tecan Group AG, Männedorf, Switzerland). Chlorophyll a, b and c were calculated following Jeffrey and Humphrey (1975), extrapolated to the total sample and normalised to the area of two tiles (i.e. µg cm^−2^).

### DNA metabarcoding

#### DNA extraction, amplification and sequencing

DNA was extracted from 0.2 g wet weight of biofilm using the DNeasy PowerBiofilm Kit (Qiagen, Hilden, Germany) following the manufacturer's instructions. DNA quantity and quality were determined photometrically with a NanoDrop 2000 (Thermo Scientific, Wilmington, United States). To assess the prokaryotic diversity, the hypervariable V4 region of the 16S rRNA gene was targeted using the primer pair 515Fm (5′-GTGYCAGCMGCCGCGGTAA-3′) and 806Rm (5′-GGACTACNVGGGTWTCTAAT-3′, Walters et al. 2016). The eukaryotic communities were investigated using the hypervariable V4 region of the 18S rRNA gene as metabarcode and the primer pair V4-TAR-EUK_F (5′-CCAGCASCYGCGGTAATTCC-3′) and V4-TAR-Euk_R (5′-CAGACTTTCGTTCTTGATYRA-3′, (Stoeck et al. 2010)). Both PCR protocols employed an initial activation step at 98°C for 30 s, followed by 26 cycles consisting of 98°C for 10 s, 63°C for 30 s and 72°C for 30 s, and a final extension for 5 min at 72°C. The three individual PCR products of the same treatment were pooled prior to sequencing. Preliminary test sequencing indicated low within-treatment variability in community composition, justifying this approach. Thereby, sequencing depth was optimized, enhancing the detection of rare taxa, while still representing the community structure of the respective treatment. Library preparation followed Next Ultra DNA Library Prep Kit for Illumina (NEB, Ipswich, United States). The quality of the final libraries was assessed with an Agilent Bioanalyzer 2100 system. Libraries were sequenced on an Illumina MiSeq platform, generating 2 × 250 bp paired-end reads by SeqIT, Kaiserslautern, Germany.

#### Sequence data processing and taxonomic assignment

Initially, excessive primer overhangs were clipped from the raw reads using cutadapt v1.18 (Martin 2011). Reads were then further processed using DADA2 v1.16 (Callahan et al. 2016) as described for hypervariable taxonomic marker genes from metabarcoding studies (Forster et al. 2019) with the following criteria: filterAndTrim with truncLen= 230 and maxEE= 1. The truncation length criterion was determined by choosing the sequence position at which Phred assigned a quality score of ≥ 30 (Q3) for at least 51% of all reads in a dataset (=base call accuracy 99.9%, (Ewing and Green 1998)). Reads were merged using 20 base pairs overlap with an allowed maximum mismatch of two and submitted to chimaera identification and removal using vsearch v2.13.7 (Rognes et al. 2016). Taxonomy was assigned to resulting amplicon sequence variants (ASVs) using the SINTAX algorithm (Edgar 2016) against the Greengenes database v13.5 (McDonald et al. 2012) for prokaryotes and the PR2 database for eukaryotes (Guillou et al. 2012). After merging the ASV-contingency table with the taxonomic information, ASVs without any taxonomic assignment and which occurred with less than five reads, and thus, may be artifactual sequences (Bokulich et al. 2013) as well as non-target (i.e. metazoa and embryophyta) ASVs were removed.

### Chemical analyses

To verify exposure concentrations, 10 ml of water was collected from the channel medium after each medium renewal throughout the study and stored at −20°C in glass scintillation vials. Concentrations were measured via TSQ Quantis plus (Thermo Fischer Scientific Inc., Waltham, United States) using matrix-matched (separate per used medium) external calibration rows (ciprofloxacin and propyzamide PESTANAL, Sigma-Aldrich, St. Louis, United States). The chromatographic separation was done using a 50×2.1 mm ThermoHypersil GOLD column (1.9 µm particle size). See details on the chemical analysis in Schreiner et al. (2020). Chemicals were quantified for only two of the four (10 & 1000 µg L^−1^ ciprofloxacin) and five (10 & 10.000 µg L^−1^ propyzamide) nominal concentrations, respectively. The measured concentrations from these levels were used to infer trends across the other concentration levels.

Chemical concentrations showed partial variations from the nominal concentrations (Table S4). Nonetheless, the measured concentrations confirmed the presence of the stressors. Despite these deviations, nominal concentrations were achieved to a sufficient extent given the high volume (40 L) in the flumes, strong evaporation, weekly medium change, metabolization and sorption by the biofilm over the course of the experiments. Since the measured concentrations generally exhibit the expected trends, the nominal concentrations are used throughout this study.

For ciprofloxacin, the 10 µg L^−1^ treatment resulted in mean measured concentrations of 18 µg L^−1^ (experiment 1) and 14 µg L^−1^ (experiment 2). The 1000 µg L^−1^ treatment was confirmed with deviations not exceeding 20% (i.e. 1200 and 990 µg L^−1^, in experiments 1 and 2, respectively). In contrast to ciprofloxacin, measured propyzamide concentrations were consistently a factor of three below nominal concentrations. In fact, the 10 and 10,000 µg L^−1^ treatments both reached 3 µg L^−1^ and 2800 µg L^−1^ on average in experiments 3 and 4, respectively.

### Statistical analyses

Differences in biofilm functional endpoints (AFDW and chlorophyll concentrations) across levels of the explanatory variables—chemical concentration and nutrients—were tested using non-parametric Kruskal-Wallis rank sum tests (Kruskal and Wallis 1952). Relative ASV abundances were calculated by dividing the reads of a respective ASV by the total sum of the reads. Weighted principal coordinates analysis (WPCA) was used on relative ASV abundances using vegan v2.6–4 (Oksanen et al. 2022), to visualise and interpret similarities and differences of the complex community data. Significances were tested with permutational multivariate analysis of variance (PERMANOVA, (Anderson 2001)). Species contribution to between-group dissimilarities on the family level was calculated with similarity percentage analysis (SIMPER). Biodiversity changes were assessed using indices including evenness, Shannon diversity, Simpson diversity, and taxa richness. Since sequencing was performed on only one pooled sample per treatment, regression-type analyses were not performed due to the limited number of data points per treatment and the increased risk of type I errors from multiple testing, and therefore, these results are presented and discussed only descriptively. To examine potential correlations between functional and structural responses, Mantel tests were performed. Raw data and code are freely available at https://doi.org/10.5281/zenodo.15520640. Metabarcoding sequence data are deposited at NCBIs SRA under the accession number: PRJNA1259222.

## Results

### Functional parameters

**Figure 1.**
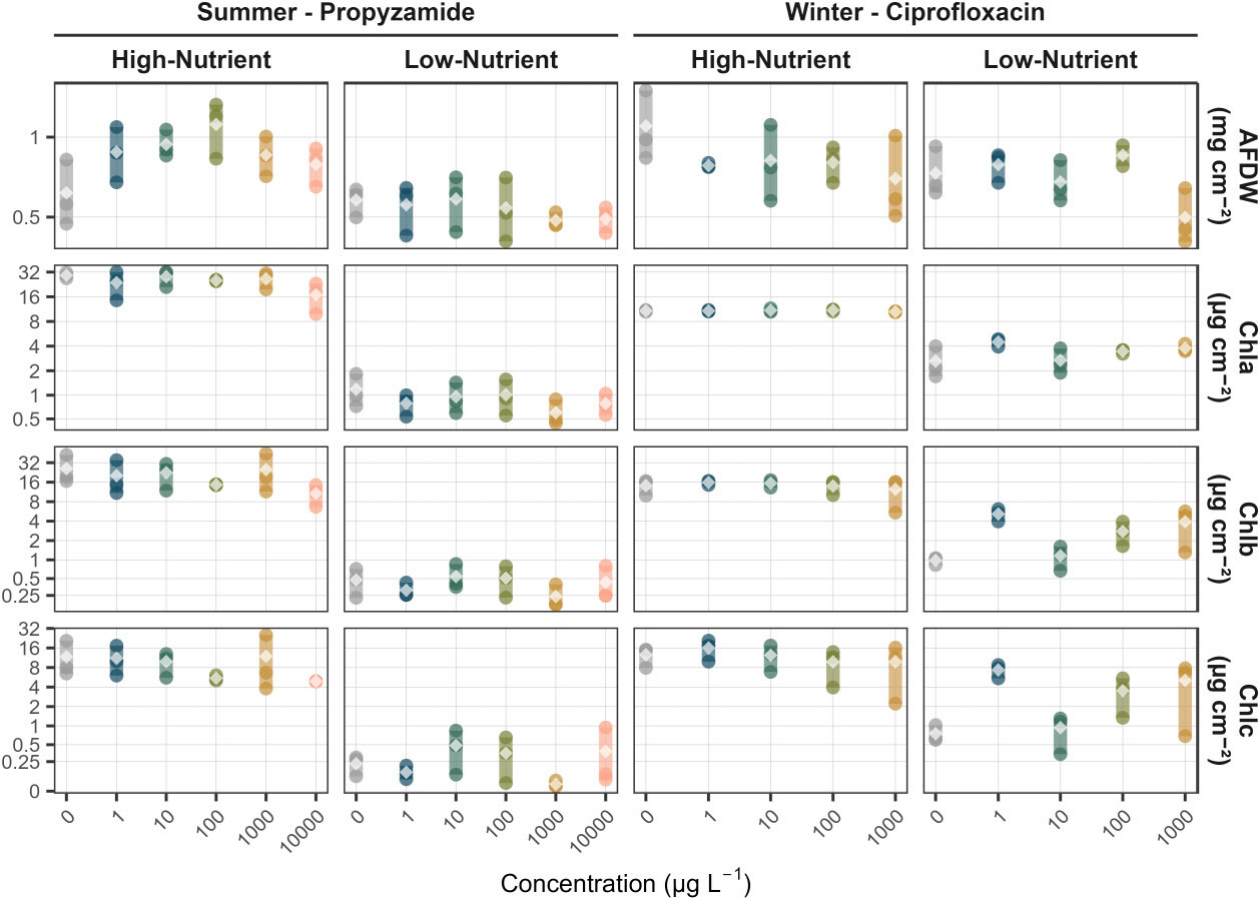
Functional parameters of freshwater biofilms ash-free dry weight (AFDW) in mg cm^−2^ and chlorophyll concentrations (Chla = chlorophyll a, Chlb = chlorophyll b, Chlc = chlorophyll c) in µg cm^− 2^ exposed to ciprofloxacin and propyzamide at different nutrient conditions (low vs. high). Data points represent individual measurements (n=3), with colours indicating different concentration levels. Mean values are shown as grey diamonds. The x-axis represents the chemical concentration (µg L^−1^) and the y-axis the measured functional parameter, both displayed on a pseudo-logarithmic scale to accommodate zero values while preserving a log-like distribution.

Despite visual trends suggesting dose-dependent responses (Fig. 1), neither AFDW nor chlorophyll a, b, or c concentrations were statistically significantly affected by chemical exposure in any of the experiments (p > 0.4 across endpoints, see Table S5). In contrast, nutrients emerged as a strong driver of biofilm functional parameters, with highly significant differences between high- and low-nutrient conditions in nearly all cases. Irrespective of the chemical exposure, AFDW significantly increased under high-nutrient conditions during summer (herbicide exposure, p < 0.001, see Table S6), but not during winter (antibiotic exposure, p = 0.19). Chlorophyll concentrations were strongly and consistently elevated under high-nutrient conditions across both seasons (p < 0.001, Fig. 1).

When comparing the control treatments, high-nutrient conditions led to a 1.5-fold increase in AFDW in winter (from 0.7 mg cm^−2^ in low-nutrient medium to 1.1 mg cm^−2^ in high-nutrient medium), whereas only a marginal increase was observed in summer (0.5 to 0.6 mg cm^−2^). Nutrient effects were substantially more pronounced in chlorophyll concentrations. Chlorophyll a increased 4-fold in winter (2.6 µg cm^−2^ in low-nutrient medium to 10.7 µg cm^−2^ in high-nutrient medium) and 25-fold in summer (1.1 to 29.2 µg cm^−2^). Chlorophyll b showed a 15-fold increase in winter (0.9 to 14.2 µg cm^−2^) and a 55-fold increase in summer (0.4 to 25.9 µg cm^−2^). Similarly, chlorophyll c increased 17-fold in winter (0.7 to 12.5 µg cm^−2^) and 51-fold in summer (0.2 to 11.6 µg cm^−2^).

No correlation was found between the functional endpoints and the community changes in each of the experiments (i.e. Mantel tests, p > 0.2, see Table S7).

### DNA metabarcoding

**Figure 2.**
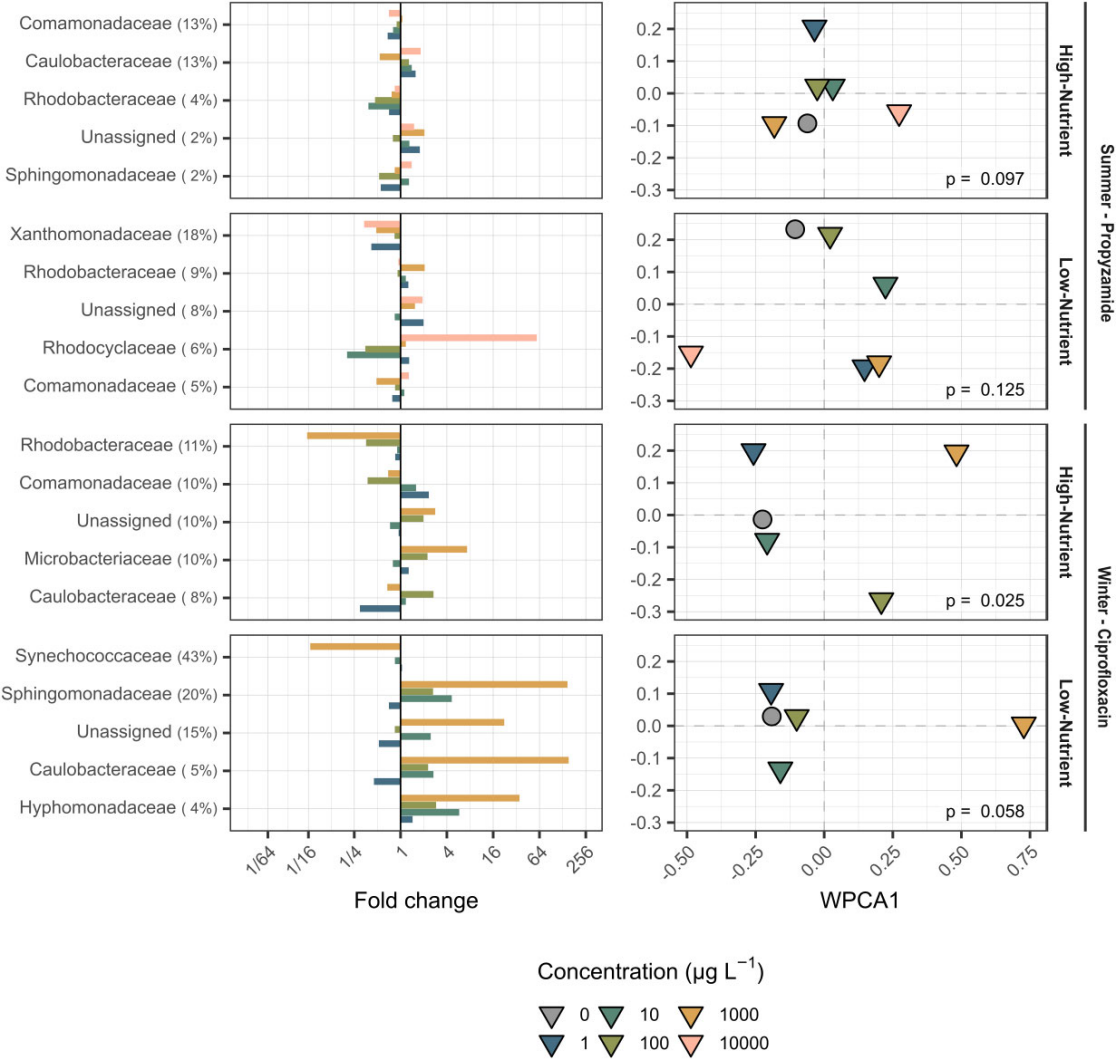
Metabarcoding-based community analysis of prokaryotes. The left column presents fold changes in relative abundance of prokaryotic families compared to the control, normalised to the mean relative abundance in the control. Taxonomic groups are ranked according to their contribution to dissimilarity between treatments and the control, chemical concentrations are differentiated by colour. The right column displays the weighted principal coordinates analysis (WPCA) plots based on relative abundances of all amplicon sequence variants (ASVs), illustrating differences in community composition. P-values indicate results from PERMANOVA. Chemical treatments are represented by triangles and differentiated in their concentration by colour, while the control is represented as a grey circle.

A total of 4887 high-quality prokaryotic ASVs were detected. Antibiotic ciprofloxacin exposure significantly altered the prokaryotic community (high-nutrient: p = 0.025; low-nutrient: p = 0.058, Fig. 2). Exposure to the herbicide propyzamide showed no significant effect, despite visually discernible differences from the control in the WPCA (high-nutrient: p = 0.097; low-nutrient: p = 0.125).

Biodiversity indices (evenness, Shannon and Simpson) increased with chemical exposure in winter under low-nutrient conditions and decreased in taxa richness in summer under high-nutrient conditions (Fig. S2). Cyanobacteria (family: Synechococcaceae) dominated the prokaryotic community in winter under low-nutrient conditions but almost vanished in the highest treatment, being replaced by Proteobacteria (family: Sphingomonadaceae) and Planctomycetes (Figs. S3 & S4). Proteobacteria (families: Comamonadaceae, Caulobacteraceae, Xanthomonadaceae and Rhodobacteraceae) were most abundant in both high-nutrient conditions as well as in summer under low-nutrient conditions and acted as main contributor to between-group dissimilarities (Fig. 2, Fig. S5).

**Figure 3.**
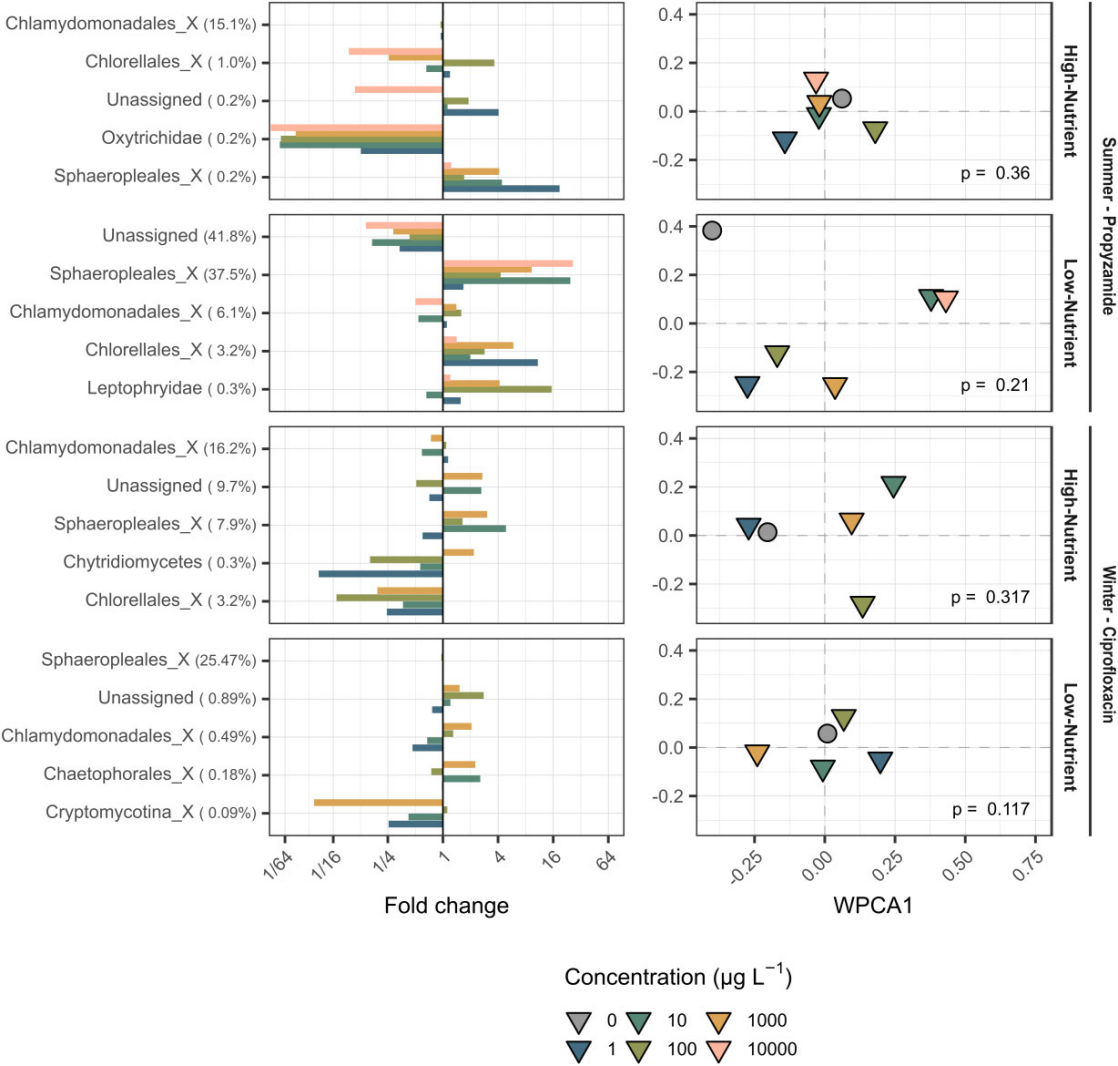
Metabarcoding-based community analysis of eukaryotes. The left column presents fold changes in relative abundance of eukaryotic families (_X: within the order of the respective family) compared to the control, normalised to the mean relative abundance in the control. Taxonomic groups are ranked according to their contribution to dissimilarity between treatments and the control, chemical concentrations are differentiated by colour. The right column displays weighted principal coordinates analysis (WPCA) plots based on relative abundances of all amplicon sequence variants (ASVs), illustrating differences in community composition. P-values indicate results from PERMANOVA. Chemical treatments are represented by triangles and differentiated in their concentration by colour, while the control is represented as a grey circle. In the propyzamide high-nutrient experiment, the relative abundance of Oxytrichidae at the highest concentration (10 000 µg L^−1^) dropped to ∼1/115 of the control (≈115-fold decrease), exceeding the fold-change axis limits and therefore not fully displayed in the figure.

A total of 8225 high-quality target eukaryotic ASVs were detected. Exposure to both antibiotic and herbicide resulted in ecologically substantial community changes relative to the control; however, these differences were not statistically significant (propyzamide: high-nutrient: p = 0.36, low-nutrient: p = 0.21; ciprofloxacin: high-nutrient: p = 0.317, low-nutrient: p = 0.117; Fig. 3).

Biodiversity indices slightly increased with ciprofloxacin exposure, despite a decreasing taxa richness in the high-nutrient experiment (Fig. S6). In both propyzamide experiments, no dose-response effects but a clearly higher diversity in terms of taxa richness and evenness in low-nutrient conditions was detected. Across all four experiments, the eukaryotic community was predominantly composed of Chlorophyta (Fig. S7 & S8), therefore acting as main dissimilarity driver between experimental treatments and the control (Fig. 3 & Fig. S9). High-nutrient conditions were primarily dominated by the green algae family Chlamydomonadales_X (within the order Chamlydomonadales), whereas low-nutrient conditions exhibited distinct community structures: after ciprofloxacin exposure, the community was dominated by Sphaeropleales_X, while a mixed composition of Sphaeropleales_X, Chlorellales_X, and Chlamydomonadales_X was detected after propyzamide exposure (Fig. S8).

## Discussion

Our study investigated the functional and structural responses of freshwater autotrophic biofilms to chronic chemical exposure (herbicide or antibiotic) under different nutrient conditions across two seasons. Ciprofloxacin and propyzamide were used as model stressors to target different domains of biofilm communities, that is prokaryotes and eukaryotes, respectively. Moreover, both contaminants reflect common exposure scenarios in freshwater ecosystems (Meffe and De Bustamante 2014, Danner et al. 2019, Liess et al. 2021). Contrary to our hypothesis (iv), functional parameters such as AFDW and chlorophyll concentrations were not significantly affected by either ciprofloxacin or propyzamide. In contrast, nutrient availability altered functional parameters more strongly. Analyses of the biofilm community composition through DNA metabarcoding revealed that chemical exposure induced shifts in its composition, suggesting a sufficient degree of functional redundancy in freshwater biofilm communities (Fetzer et al. 2015).

### Toxicity of ciprofloxacin to freshwater biofilms

The antibiotic ciprofloxacin significantly altered the structure of the prokaryotic community (hypothesis i) in winter, particularly under high-nutrient conditions. This confirms previous findings that fluoroquinolones (the antibiotic class to which ciprofloxacin belongs) strongly affect bacterial diversity (Johansson et al. 2014). The observed increase of the biodiversity indices (evenness, Shannon and Simpson; Fig. S2) under low-nutrient conditions likely reflects a release from competitive exclusion, allowing more tolerant or previously suppressed taxa to proliferate (Romero et al. 2020). Cyanobacteria (Synechococcaceae) were prevalent under low-nutrient conditions but almost vanished at high chemical concentrations. Their disappearance was paralleled by a relative increase in Proteobacteria, especially Sphingomonadaceae—a group known for its metabolic versatility and ability to degrade xenobiotics (Glaeser and Kämpfer 2014). Similarly, the proliferation of Planctomycetes—taxa involved in carbon and nitrogen cycling—may indicate the exploitation of xenobiotic compounds as alternative carbon sources under nutrient-limited conditions. Despite these community shifts, no significant effects on AFDW or chlorophyll concentrations were observed over the four 14-day experiments. This resilience likely reflects the high microbial redundancy in biofilm communities, where altered community structure can sustain core functions (Besemer 2015, Philippot et al. 2021). To further evaluate correlations between structural and functional responses, Mantel tests were performed between community composition and functional endpoints. No significant correlations were detected in each of the experiments, supporting the interpretation that structural shifts did not translate into measurable functional changes.

### Toxicity of propyzamide to freshwater biofilms

Despite statistically non-significant effects of the eukaryotic community, ecologically significant effects were observed, especially at the family level (hypothesis i). However, functional endpoints remained unaffected, even at the highest concentrations tested. This structural-functional disconnection suggests a decoupling of community composition and ecosystem processes, driven by biofilms’ functional redundancy. Chlorophyta remained dominant across treatments (Fig. S7), though changes in relative abundances at the family level (e.g. from Chlamydomonadales_X to Sphaeropleales_X in the low-nutrient medium; Fig. S8) indicated a treatment-related shift in community composition—possibly reflecting differential tolerance among taxa. These findings align with prior work showing that herbicides like diuron and diflufenican can significantly alter algal community composition without necessarily impairing overall photosynthetic activity (Ricart et al. 2009, Feckler et al. 2018). In high-nutrient media, AFDW increased slightly with a maximum at 100 µg L^−1^ (Fig. 1), suggesting a hormetic effect (Schmitt-Jansen and Altenburger 2005), which was not reflected by changes in the community (Fig. S7 & S8).

The absence of dose-dependent trends across functional and structural endpoints suggests potential threshold or a non-monotonic biofilm response to propyzamide. This is consistent with findings from both field and mesocosm studies, where community responses to herbicide mixtures often display non-monotonic patterns (Rydh Stenström et al. 2021). Biofilms may buffer low-level stress through shifts in taxonomic composition, with only subtle functional consequences until a disturbance threshold is exceeded. In this context, low to moderate (µg L^−1^) propyzamide exposure may facilitate the coexistence of tolerant and sensitive taxa, while higher concentrations could lead to selective exclusion and compositional simplification.

### Nutrient effects

Nutrient availability, rather than chemical stress, was the dominant factor influencing biofilm functional responses (hypothesis iv). Biofilms colonised in high-nutrient conditions exhibited significantly greater biomass supporting several earlier reports (Dodds & Smith, 2016; Mulholland et al. 1991). Notably, the magnitude of this response was seasonal, with more pronounced increases in biomass and chlorophyll concentrations observed during the ciprofloxacin experiments in winter. This suggests that nutrient limitation is more severe in winter biofilms, likely due to reduced metabolic rates associated with lower temperatures, slower colonisation and diminished light availability during colder months (Rosemond et al. 2000), which is partially incorporated by the lower (about 3°C) laboratory temperatures during these experiments. Notably, elevated conductivity in the high-nutrient medium may have imposed non-negligible salt stress.

Importantly, high-nutrient conditions not only increased biofilm biomass but also reshaped community composition (hypothesis ii & iii) and potential nutritional quality for grazers. As shown in previous studies, phosphorus enrichment can shift biofilm communities from diatom-dominated to chlorophyta- and cyanobacteria-dominated assemblages (Iannino et al. 2020). Since these groups are generally less rich in essential polyunsaturated fatty acids, such changes can reduce resource quality for higher trophic levels and may obscure chemically induced stress responses for primary consumers. Nutrients can modulate the effects of other environmental stressors through both direct physiological support (e.g. enhanced growth or energy availability) and community-level shifts (Bundschuh et al. 2020, Romero et al. 2020). Biofilms colonised under high-nutrient conditions may exhibit increased growth rates and altered community structure, potentially leading to modified resilience against stressors. Rindi and Benedetti-Cecchi (2023) found that nitrogen and phosphorus enrichment—regardless of concentration or combination—increased rocky intertidal biofilm sensitivity (as indicated by elevated biomass and physiological activity), while resilience remained unaffected. This nutrient-driven modulation of stress sensitivity aligns with predictions from the DEB theory (Kooijman 2010), which states that the energy available to an organism or community governs its capacity to allocate resources to growth, maintenance, and stress responses. Under high-nutrient conditions, biofilms may have greater energetic capacity to buffer chemical stress, though this may also select for fast-growing, generalist taxa with reduced ecological sensitivity. Our results highlight the importance of careful consideration given to nutrient supply, as it strongly mediates biofilm development, composition, and resilience. Artificial media with elevated nutrient conditions, though useful for promoting biomass growth, may unintentionally select for fast-growing, stress-tolerant taxa that do not adequately represent the sensitivity of natural communities towards chemical stress.

When transferring biofilms from natural habitats to the laboratory, key environmental parameters such as light, temperature, flow, and grazing pressure are modified, which inevitably shifts community composition (Muñoz et al. 2001, Wendt-Rasch et al. 2003) and potentially their stress response. This is confirmed in our study, where biofilms sampled directly from the stream were dominated by Proteobacteria, Acidobacteria, Actinobacteria, Cyanobacteria among prokaryotes (Fig. S10), and Rhodophyta, Chlorophyta and Ochrophyta among eukaryotes (Fig. S11)—typical constituents of stream biofilms (Wu 2017). In contrast, laboratory-grown communities were primarily composed of Chlorophyta (Fig. 3, Fig. S7), Proteobacteria and Cyanobacteria (Fig. 2, Fig. S3). The pronounced differences between the bacterial communities of field (Fig. S10) and laboratory-grown (Fig. S7 & S8) biofilms are partially a function of the media. The media can indeed promote algal and cyanobacterial dominance when rich in P (Iannino et al. 2020), potentially reducing competitive niches for heterotrophic bacteria and shifting overall biofilm community composition. Moreover, seasonal differences in taxa richness and diversity were evident, with higher prokaryotic and eukaryotic diversity observed in summer, likely driven by increased resource availability (Rosemond et al. 2000, Qin et al. 2007). Interestingly, eukaryotic diversity (taxa richness) in laboratory samples, especially under low-nutrient conditions, exceeded that of field samples, possibly reflecting a release from natural competition and predation. Consequently, a direct extrapolation from laboratory studies to the field, as with any laboratory study, should be interpreted with caution.

## Conclusion

Although ciprofloxacin (antibiotic) concentrations in this study exceeded most environmentally realistic concentrations, these levels are sufficient to induce community shifts, suppress sensitive taxa, and promote resistance (Martins et al. 2012), with potential bottom-up effects on trophic interactions and ecosystem functioning (Sabater et al. 2007, Konschak et al. 2020). Additionally, propyzamide concentrations tested here (up to 10 000 µg L^−1^) exceeded those typically found in surface waters, such as 0.58 µg L^−1^ reported by Liess et al. (2021). However, such high exposure levels are useful to test resistance and resilience limits and reveal potential community-level tolerance mechanisms (Tlili et al. 2016), where exposure history or selection under artificial lab conditions—particularly in high-nutrient media—favours tolerant taxa. Thus, even novel stressors may produce limited functional responses due to pre-adapted communities. In summary, while chemical exposure—for the antibiotic ciprofloxacin—induced clear structural responses in biofilm communities, functional stability was maintained, underscoring the ecological buffering capacity of stream biofilms. However, the 14-day exposure duration may not capture long-term effects or delayed functional impacts, and further research is needed to assess temporal dynamics in biofilm functioning as well as its potential to recover.

## Supplementary Material

fiaf094_Supplemental_File
