# Supplementary material for: Functional stability despite structural changes in freshwater biofilm communities exposed to an antibiotic and an herbicide - the role of nutrient conditions
Source: FEMS Microbiol Ecol. 2025 Sep 24;101(10):fiaf094. doi: 10.1093/femsec/fiaf094 (PMC12481197; doi:10.1093/femsec/fiaf094)
Supplement: fiaf094_Supplemental_File [file fiaf094_supplemental_file.docx]

Supplementary material for:

**Functional stability despite structural changes in freshwater biofilm communities exposed to an antibiotic and an herbicide - the role of nutrient conditions**

Sophie Oster^1^, Eric Bollinger^1^, Verena C. Schreiner^2,3^, Tobias Schmitt^1^, Sabine Filker^4^, Mirco Bundschuh^1,5^

^1^iES Landau, Institute for Environmental Sciences, University of Kaiserslautern-Landau (RPTU), Fortstraße 7, 76829 Landau, Germany

^2^Faculty of Biology, University of Duisburg-Essen, Universitätsstraße 2, 45141 Essen, Germany

^3^Research Center One Health Ruhr, University Alliance Ruhr, Universitätstraße 2, 45141 Essen, Germany

^4^Faculty of Biology, Department of Ecology, University of Kaiserslautern-Landau (RPTU), Erwin-Schrödinger-Straße 14, 67663 Kaiserslautern, Germany

^5^Department of Aquatic Sciences and Assessment, Swedish University of Agricultural Sciences, Lennart Hjelms väg 9, SE-756 51 Uppsala, Sweden


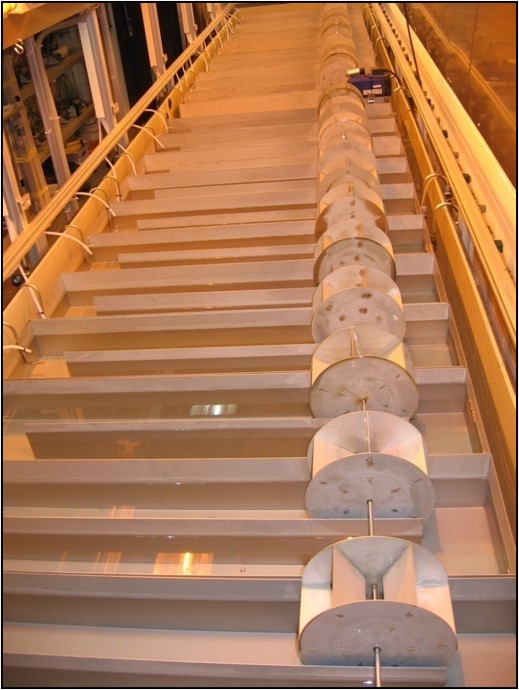


Figure S1: Landau Laboratory Stream Microcosm facility. Consists of 24 stainless-steel channels (120 x 30 x 20 cm) in total, each connected to a stainless-steel paddle wheel rotating 24 hours and creating a flow velocity of around 0.02 m s^-1^. Credit: Unknown.

Table S1: Dates and colonization times of the four experiments. Chemical-free colonization phase varied throughout the experiments, due to the difference in nutrients and daylight regime, which directly affect growth, resulting in a faster biofilm development under high-nutrient conditions and in summer. Chemical exposure always lasted for 14 days.

| Experiment | Date | Chemical free colonization phase | Nutrients | Chemical Stressor |
| --- | --- | --- | --- | --- |
| 1 | 10.02.2021 - 34.04.2021 | 8 weeks | Low | Ciprofloxacin |
| 2 | 11.02.2022 - 26.03.2022 | 4 weeks | High | Ciprofloxacin |
| 3 | 10.09.2021 - 15.10.2021 | 4 weeks | Low | Propyzamide |
| 4 | 10.08.2021 - 07.09.2021 | 2 weeks | High | Propyzamide |

Table S2: Composition of modified SAM-S5 media (Borgmann, 1996). Salts in bold letters display the modifications (Rybicki et al., 2012; Rybicki & Jungmann, 2018).

| Salt | Quantity (mg L^-1^) |
| --- | --- |
| NaHCO_3_ | 85.55 |
| KCl | 3.8 |
| NaBr | 1.03 |
| MgSO_4_x7H_2_O | 61.5 |
| CaCl_2_x2H_2_O | 147 |
| NaNO_3_ | **9** |
| Na_2_SiO_3_x5H_2_O | **5** |
| Na_2_HPO_4_ | **0.03** |

Table S3: Composition of Kuhl media (Kuhl & Lorenzen, 1964)

| Salt | Quantity (mg L^-1^) |
| --- | --- |
| KNO_3_ | 1011.1 |
| NaHPO_4_ x H_2_O | 621 |
| Na_2_HPO_4_ | 71 |
| MgSO_4_ x 7H_2_O | 246.5 |
| CaCl_2_ x 2H_2_O | 14.7 |
| FeSO_4_ x 2H_2_O | 7 |
| Na_2_EDTA x H_2_O | 9.3 |
| H_3_BO_3_ | 0.061 |
| MnSO_4_ x H_2_O | 0.169 |
| ZnSO_4_ x 7H_2_O | 0.287 |
| CuSO_4_ x 5H_2_O | 0.0025 |
| Na_2_MoO_4_ x 2H_2_O | 0.017 |

Table S4: Mean measured concentrations of chemical stressors after medium change for each experiment. The limit of quantification of ciprofloxacin and propyzamide was 7.5 µg L^-1^ and 0.5 µg L^-1^, respectively.

| Experiment | Treatment | Nominal concentration (µg L^-1^) | Mean  measured concentration (µg L^-1^) |
| --- | --- | --- | --- |
| 1 | Ciprofloxacin | 10 | 18.38 |
| 1 | Ciprofloxacin | 1000 | 1200 |
| 2 | Ciprofloxacin | 10 | 14.42 |
| 2 | Ciprofloxacin | 1000 | 988.33 |
| 3 | Propyzamide | 10 | 3.63 |
| 3 | Propyzamide | 10000 | 2550.00 |
| 4 | Propyzamide | 10 | 3.23 |
| 4 | Propyzamide | 10000 | 3108.33 |

Table S5: Summary of statistical test results for AFDW and chlorophyll a, b, and c concentrations across chemical exposure treatments of the four experiments. Despite visual trends (Figure 1), no statistically significant effects were detected for any endpoint (all p > 0.4).

| Experiment | Chemical Stressor | AFDW | Chla | Chlb | Chlc |
| --- | --- | --- | --- | --- | --- |
| 1 | Ciprofloxacin | 0.425 | 0.425 | 0.420 | 0.420 |
| 2 | Ciprofloxacin | 0.604 | 0.554 | 0.798 | 0.757 |
| 3 | Propyzamide | 0.798 | 0.681 | 0.672 | 0.425 |
| 4 | Propyzamide | 0.425 | 0.504 | 0.504 | 0.425 |

Table S6: Summary of statistical test results for AFDW and chlorophyll a, b, and c concentrations across different nutrient conditions between the two experiments with the same chemical stressor.

| Experiments | Chemical Stressor | AFDW | Chla | Chlb | Chlc |
| --- | --- | --- | --- | --- | --- |
| 1&2 | Ciprofloxacin | 0.190 | 0.00000613 | 0.00000732 | 0.0000852 |
| 3&4 | Propyzamide | 0.0000146 | 0.000000792 | 0.000000792 | 0.000000792 |

Table S7: To examine potential correlations between functional and structural responses Mantel tests were performed for each experiment separately with prokaryote and eukaryote data, respectively.

| Experiment | Prokaryotes p-value | Eukaryotes p-value |
| --- | --- | --- |
| 1 | 0.5 | 0.34 |
| 2 | 0.65 | 0.35 |
| 3 | 0.36 | 0.28 |
| 4 | 0.23 | 0.21 |

##
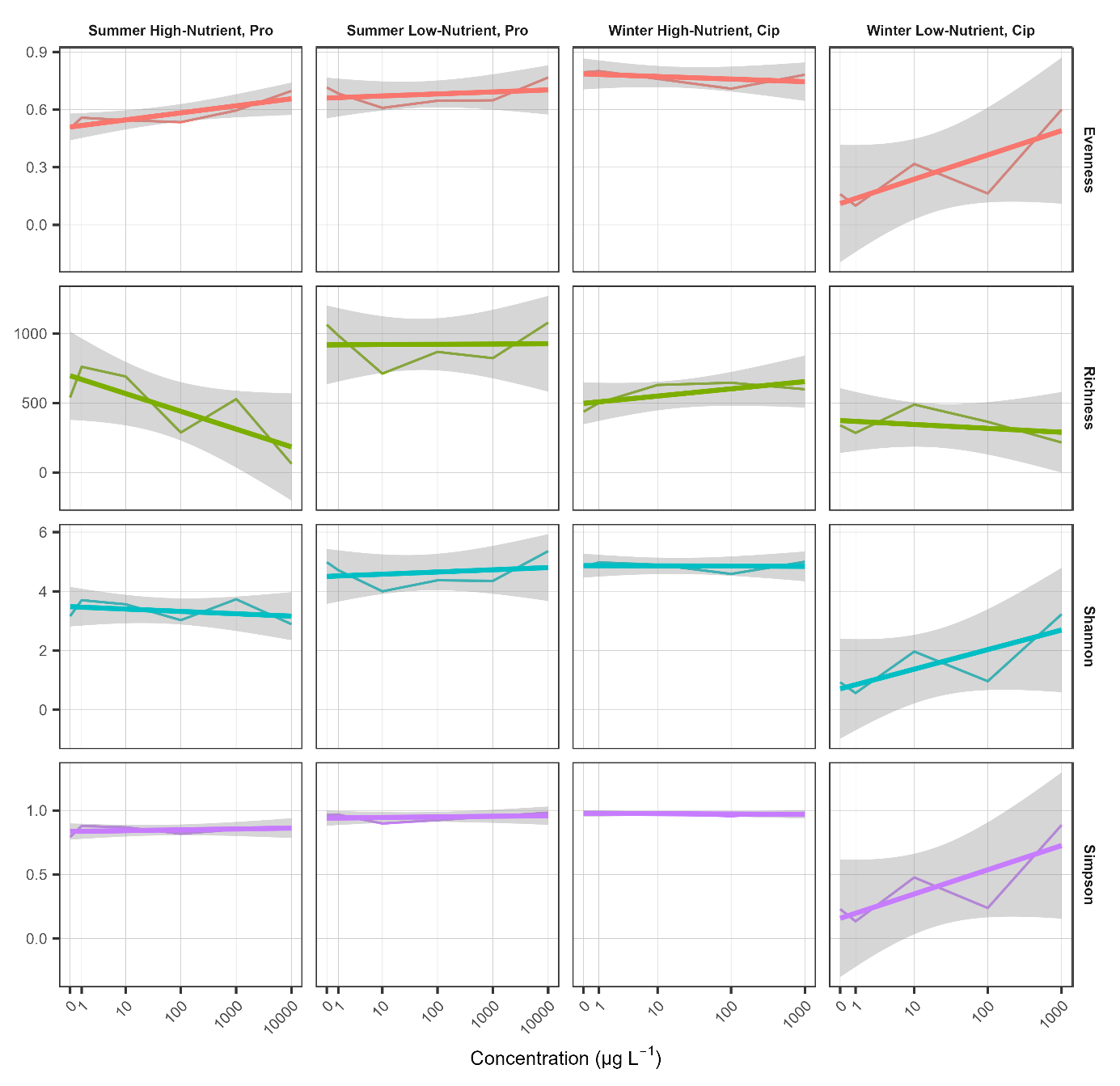


Figure S2: Relationship between prokaryotic diversity indices and chemical concentration across all four different experiments. The x-axis represents the concentration of the respective chemical (µg L⁻¹) on a pseudo-logarithmic scale. Each panel corresponds to a different diversity index (i.e., Evenness, Richness, Shannon and Simpson) and experimental setup, as indicated by the facet labels. Coloured lines represent observed data trends, while solid lines indicate linear model fits with shaded confidence intervals. The y-axis scale is adjusted independently for each diversity index to reflect variation across experiments.


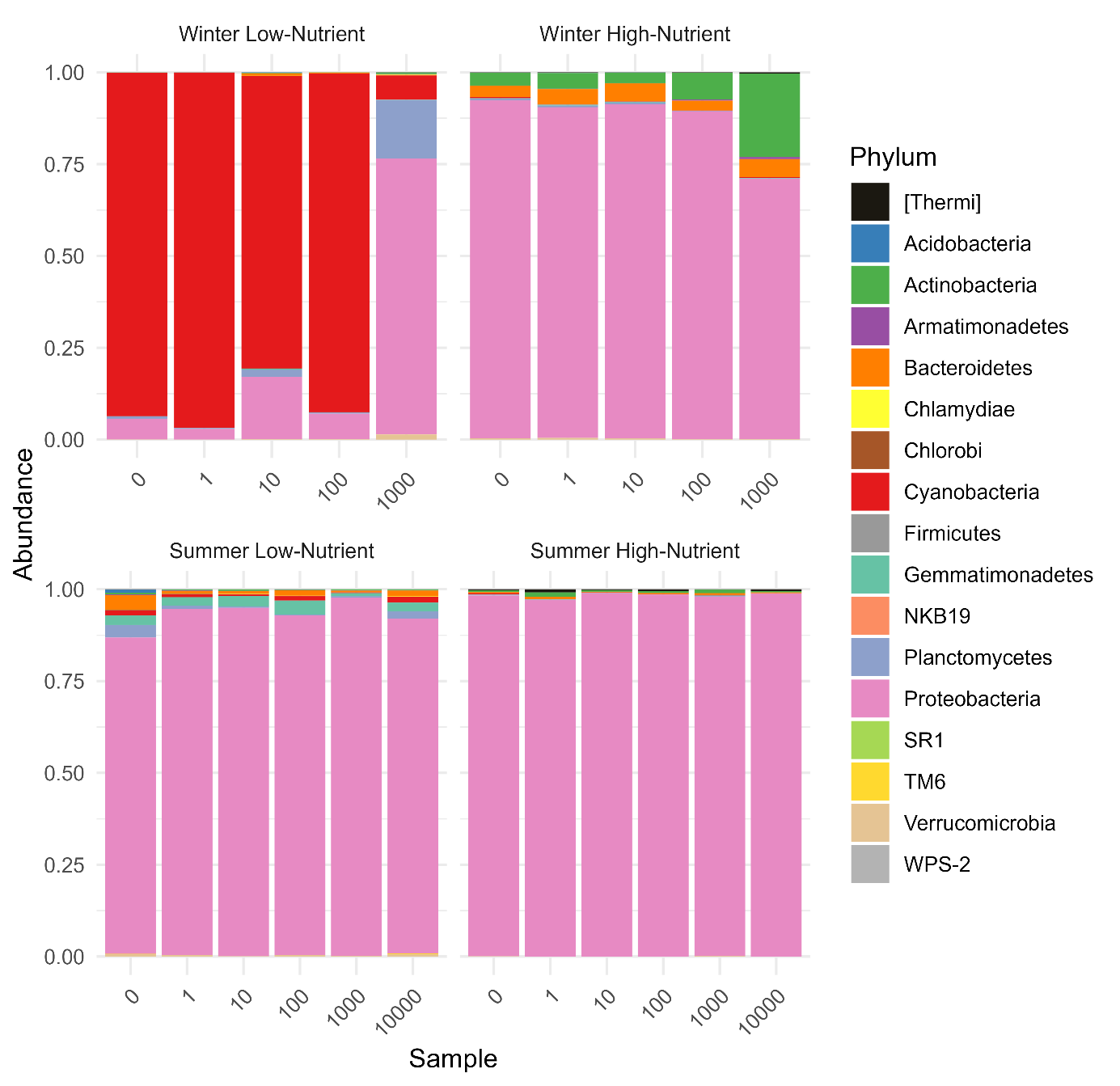
Figure S3: Composition of prokaryotic communities at the phylum level across all experimental conditions. Bars represent the relative abundance of different phyla within each sample, with colours indicating distinct taxonomic groups. Samples are grouped by experimental conditions (faceted by experiment) and are arranged along the x-axis according to increasing chemical concentration (µg L⁻¹). The x-axis labels indicate the corresponding chemical concentrations for each sample. The y-axis represents the relative abundance of each phylum.

##
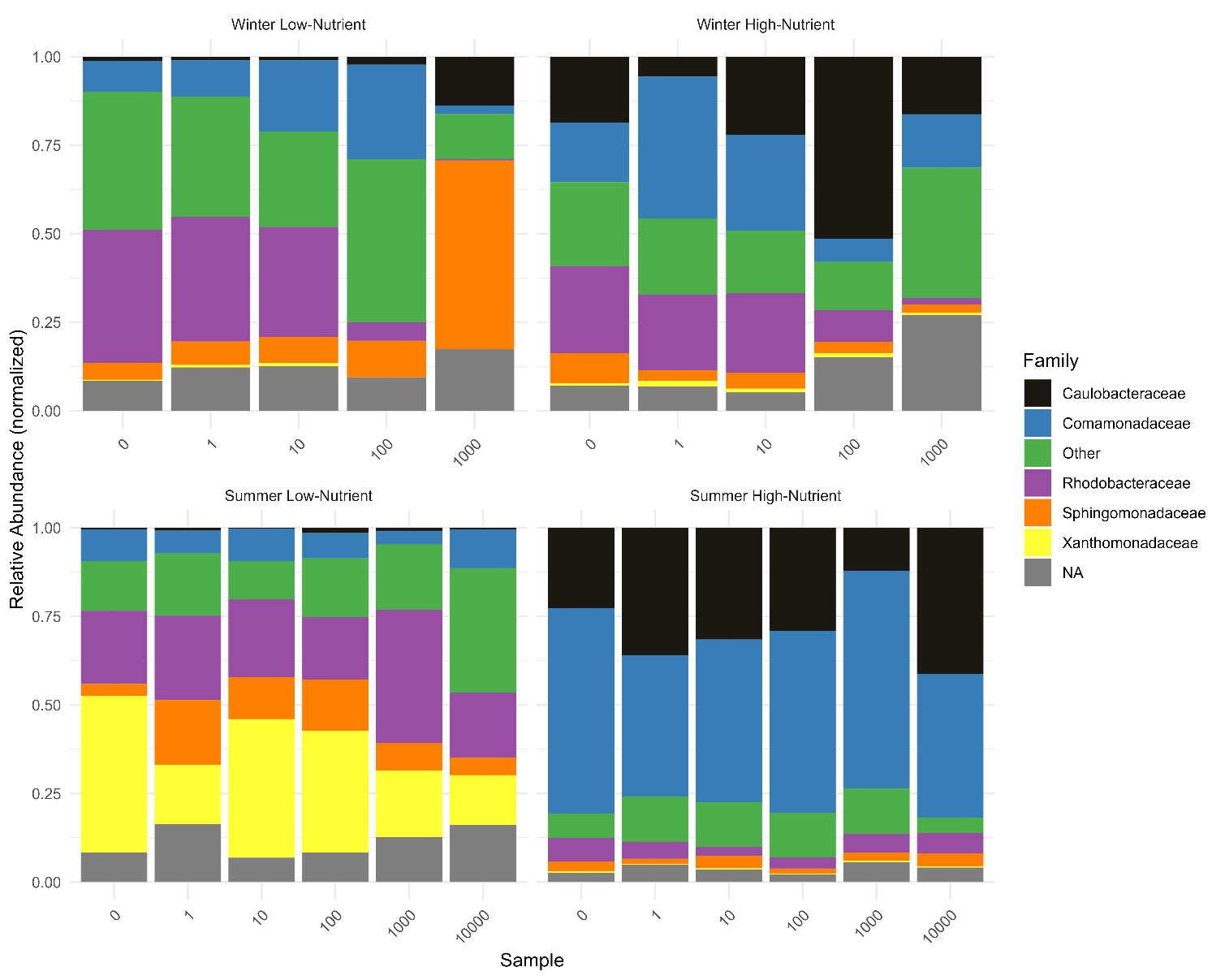


Figure S4: Composition of prokaryotic communities at the family level of Proteobacteria across all experimental conditions. Only the six most abundant families (summed across all samples) are shown while the remaining groups are displayed as “Other.” Bars represent the relative abundance of these families within Proteobacteria, normalized that the total Proteobacteria abundance sums to 1 in each sample. Colours indicate distinct taxonomic groups. Samples are grouped by experimental conditions (faceted by experiment) and arranged along the x-axis according to increasing chemical concentration (µg L⁻¹).


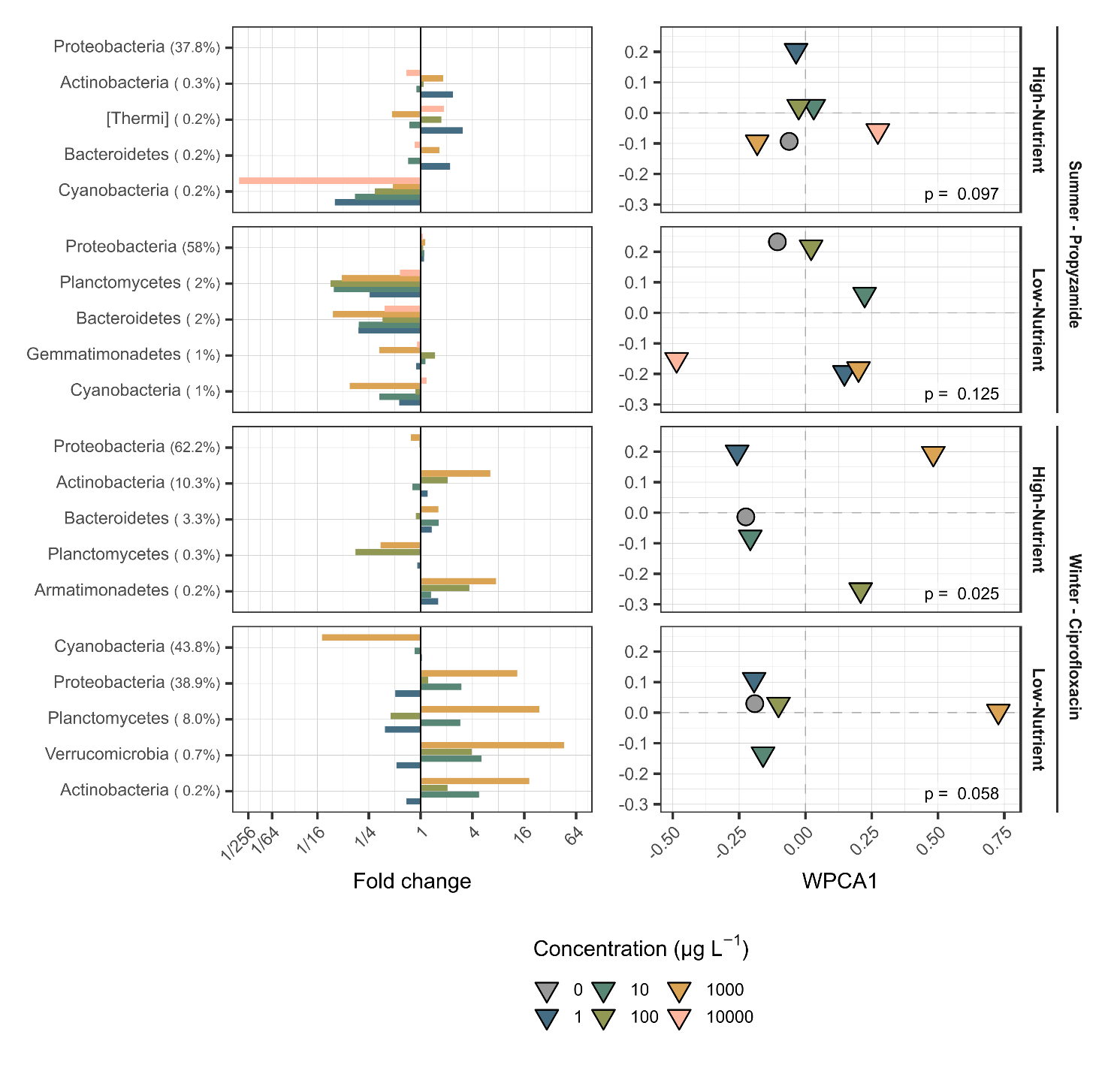


Figure S5: Metabarcoding-based community analysis of prokaryotes. The left column presents fold changes in relative abundance of prokaryotic phyla compared to the control, normalised to the mean relative abundance in the control. Taxonomic groups are ranked according to their contribution to dissimilarity between treatments and the control, chemical concentrations are differentiated by colour. The right column displays the weighted principal coordinates analysis (WPCA) plots based on relative abundances of all amplicon sequence variants (ASVs), illustrating differences in community composition. P-values indicate results from PERMANOVA. Chemical treatments are represented by triangles and differentiated in their concentration by colour, while the control is represented as a grey circle. Cyanobacteria were not present in the highest treatment of the propyzamide high-nutrient experiment, and therefore the respective bar exceeds the x-axis in the figure.

##

##
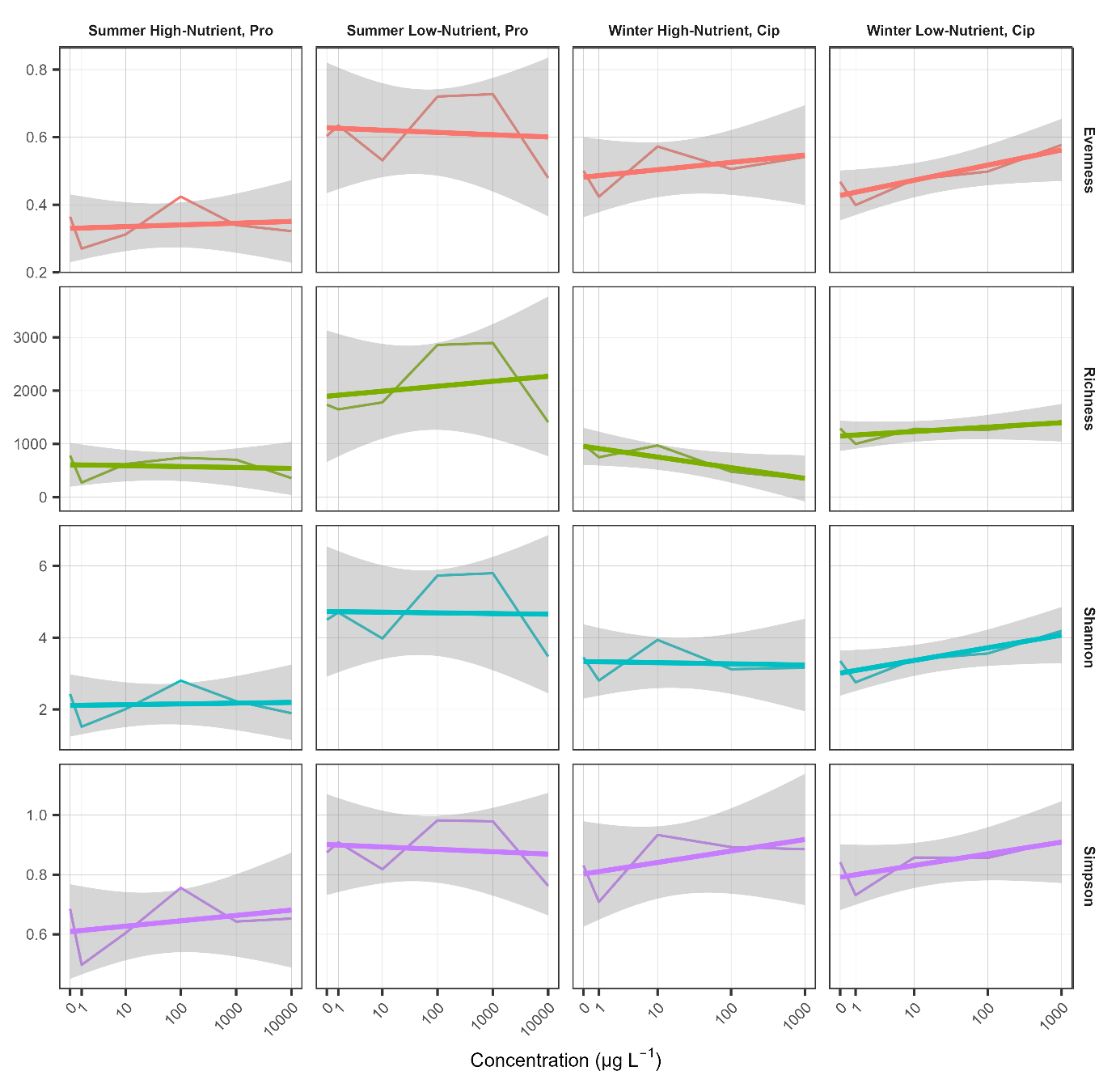


Figure S6: Relationship between eukaryotic diversity indices and chemical concentration across all four different experiments. The x-axis represents the concentration of the respective chemical (µg L⁻¹) on a pseudo-logarithmic scale. Each panel corresponds to a different diversity index (i.e., Evenness, Richness, Shannon and Simpson) and experimental setup, as indicated by the facet labels. Coloured lines represent observed data trends, while solid lines indicate linear model fits with shaded confidence intervals. The y-axis scale is adjusted independently for each diversity index to reflect variation across experiments.


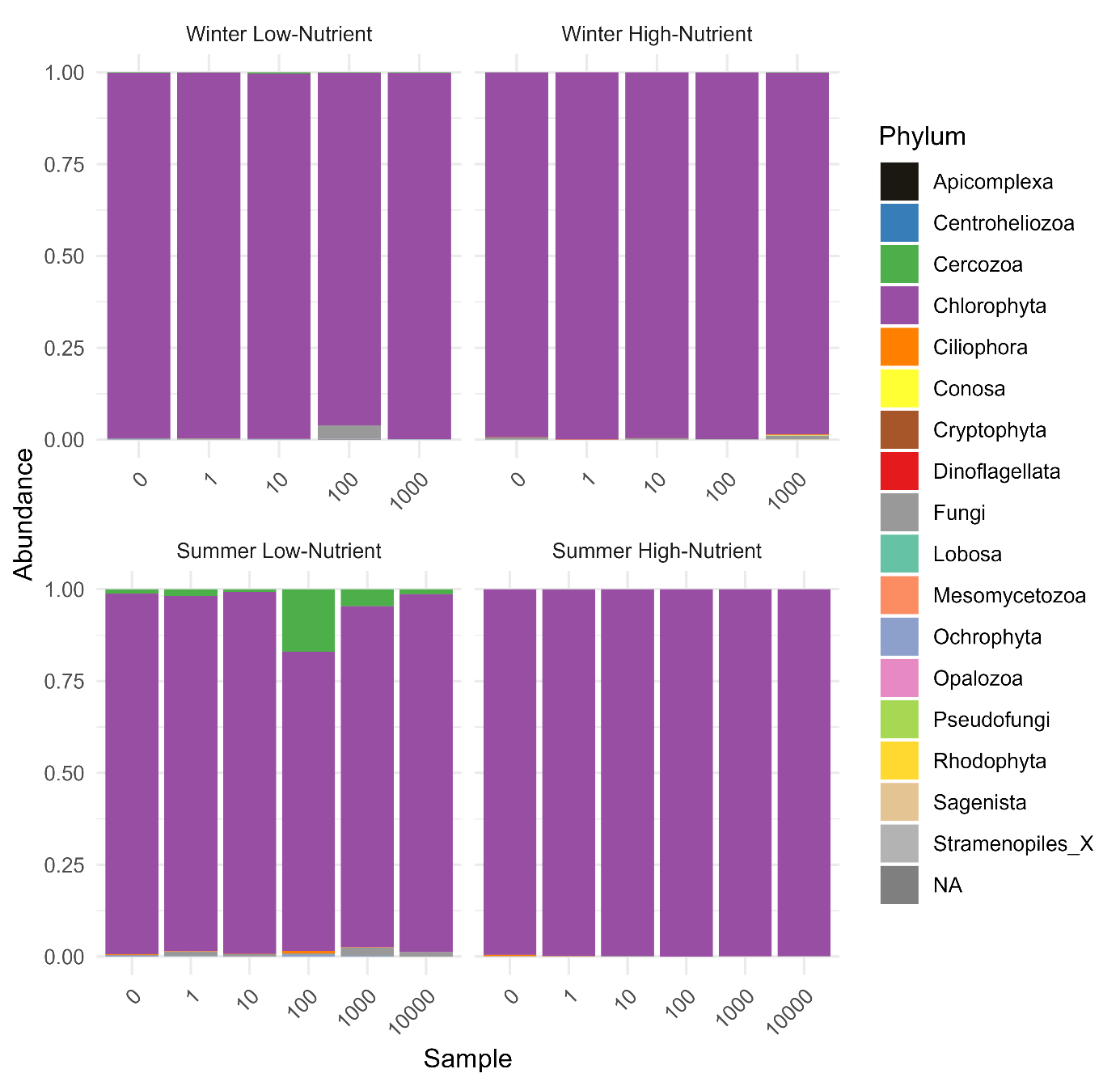


Figure S7: Composition of eukaryotic communities at the phylum level across all experimental conditions. Bars represent the relative abundance of different phyla within each sample, with colours indicating distinct taxonomic groups. Samples are grouped by experimental conditions (faceted by experiment) and are arranged along the x-axis according to increasing chemical concentration (µg L⁻¹). The x-axis labels indicate the corresponding chemical concentrations for each sample. The y-axis represents the relative abundance of each phylum.

##
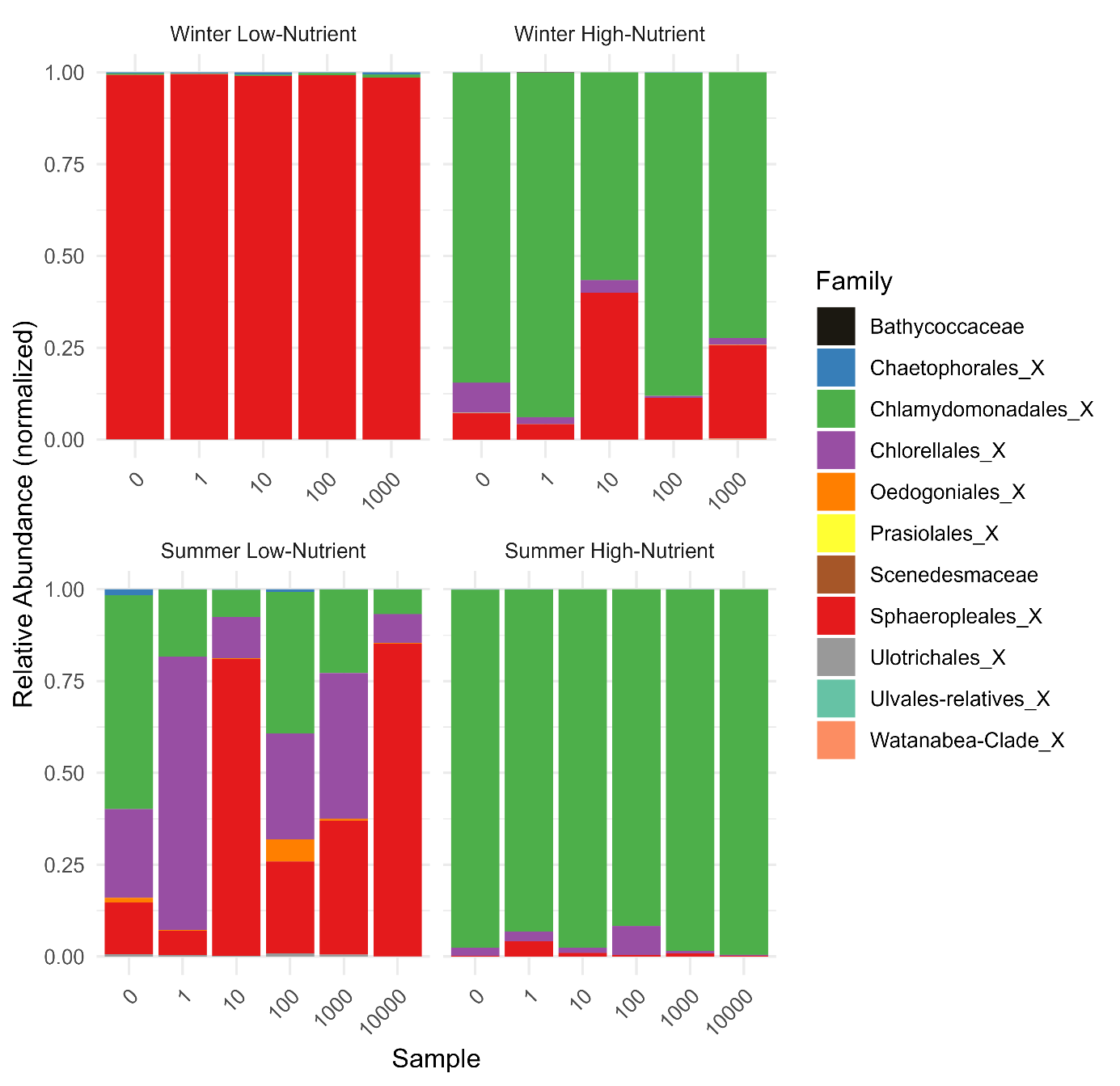


Figure S8: Composition of eukaryotic communities at the family level of Chlorophyta across all experimental conditions. Bars represent the relative abundance of different Chlorophyta families within each sample, normalized so that the total Chlorophyta abundance sums to 1 per sample. Colours indicate distinct taxonomic groups. Samples are grouped by experimental conditions (faceted by experiment) and arranged along the x-axis according to increasing chemical concentration (µg L⁻¹).

##
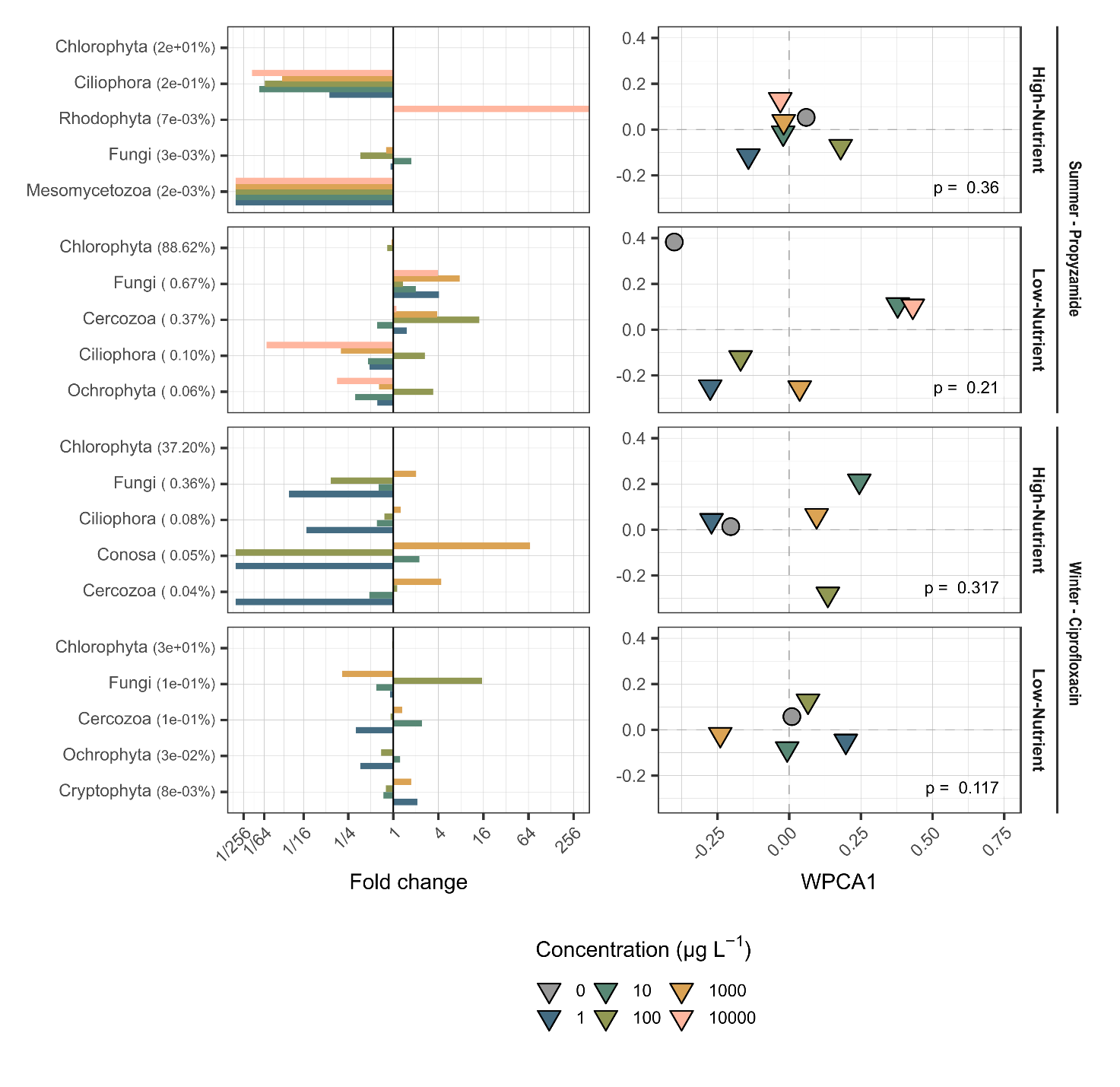


Figure S9: Metabarcoding-based community analysis of eukaryotes. The left column presents fold changes in relative abundance of eukaryotic phyla compared to the control, normalised to the mean relative abundance in the control. Taxonomic groups are ranked according to their contribution to dissimilarity between treatments and the control, chemical concentrations are differentiated by colour. The right column displays weighted principal coordinates analysis (WPCA) plots based on relative abundances of all amplicon sequence variants (ASVs), illustrating differences in community composition. P-values indicate results from PERMANOVA. Chemical treatments are represented by triangles and differentiated in their concentration by colour, while the control is represented as a grey circle. The bars in this figure that extend beyond the limits of the x-axis originate either from eukaryotic phyla that were not detected in the chemical treatments (Mesomycetozoa in the propyzamide high-nutrient experiment; Conosa and Cercozoa in the ciprofloxacin high-nutrient experiment) or were detected in the chemical treatment and not in the respective control (Rhodophyta, propyzamide high-nutrient experiment). Since no fold change can be calculated in these cases, these bars extend beyond the limits of the x-axis.

##
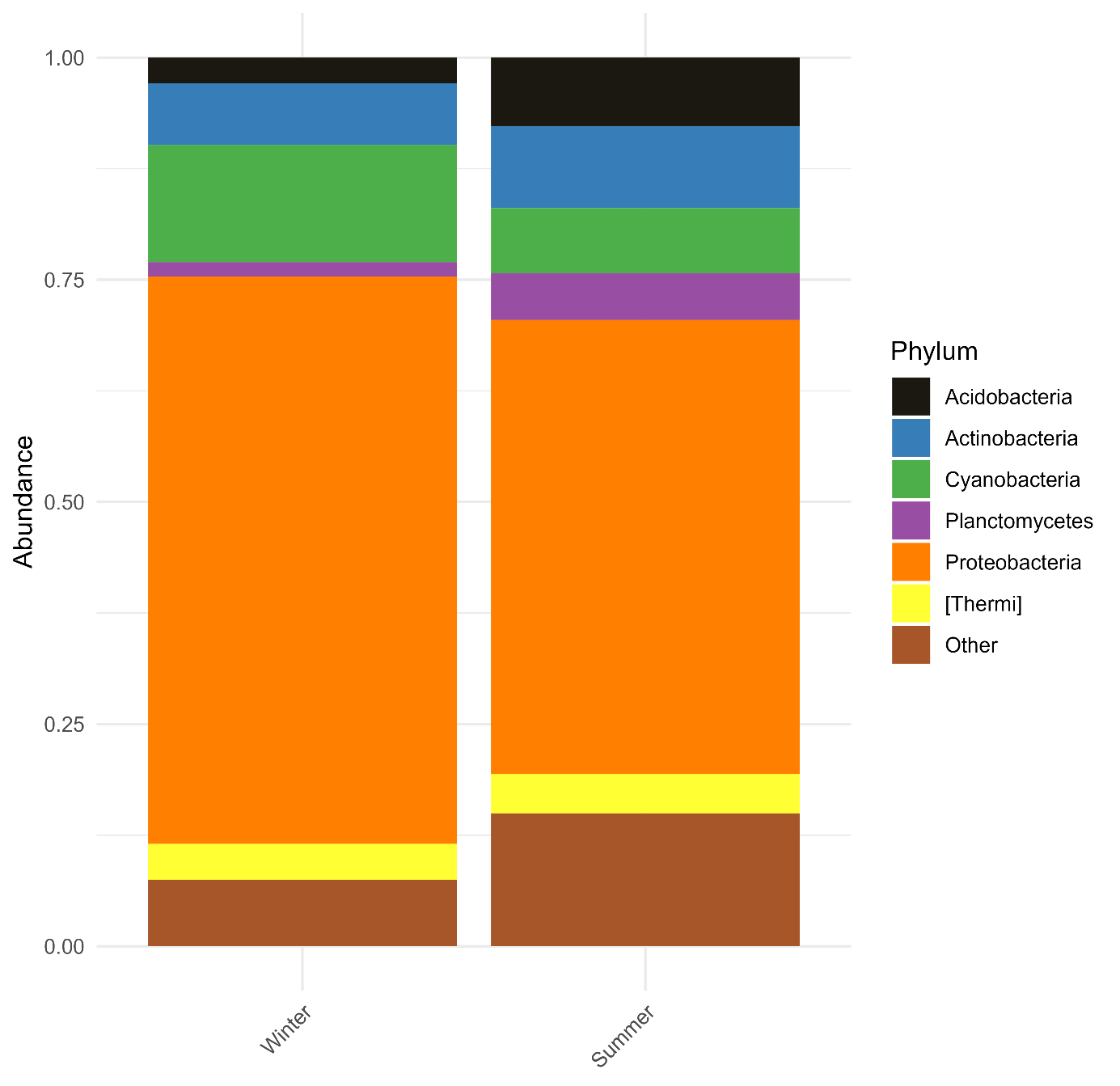


Figure S10: Composition of prokaryotic field communities in winter and summer at the phylum level (only 6 most abundant phyla summed across both samples shown). Bars represent the relative abundance of different phyla, with colours indicating distinct taxonomic groups.

##
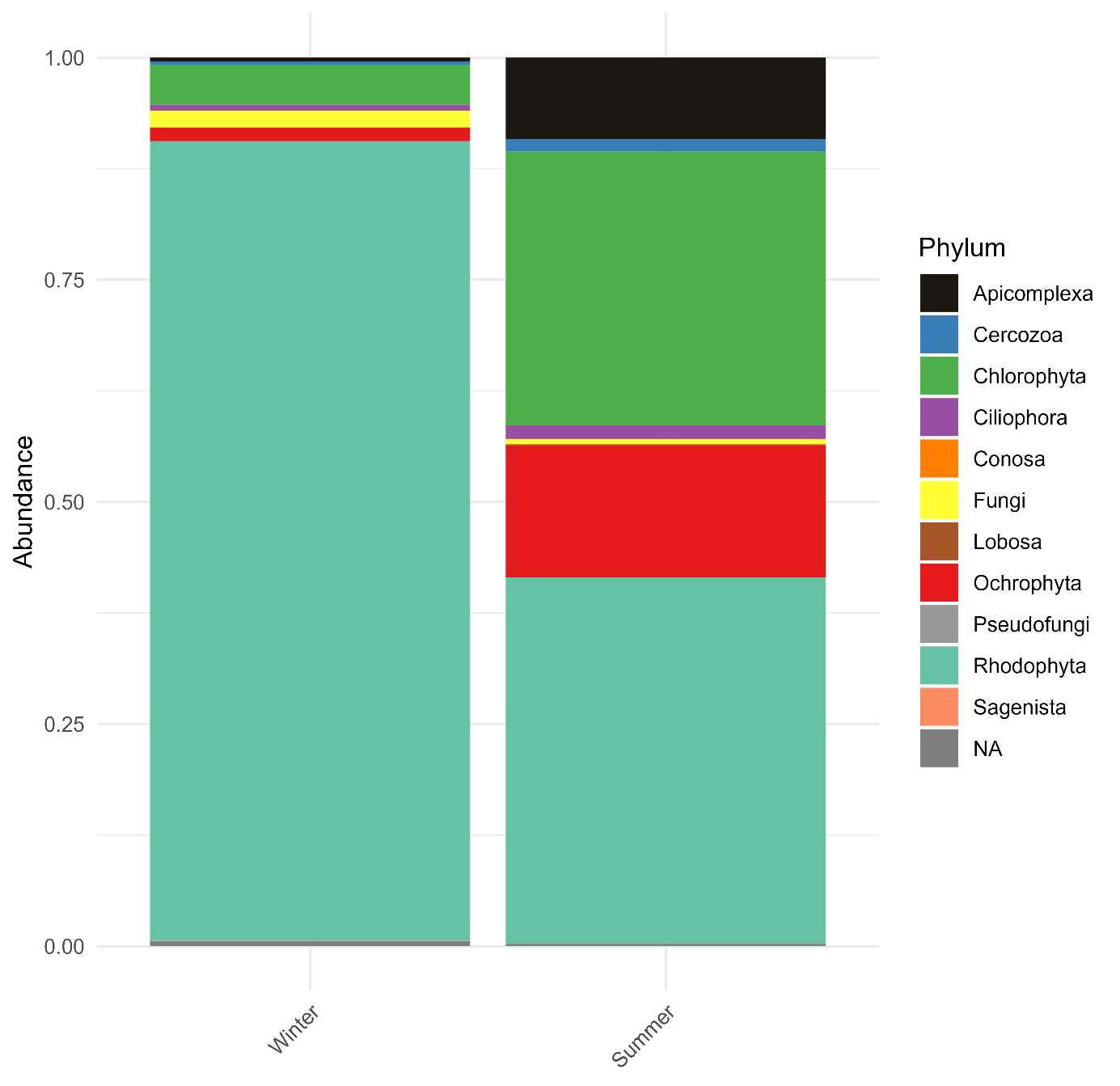


Figure S11: Composition of eukaryotic field communities in winter and summer at the phylum level. Bars represent the relative abundance of different phyla, with colours indicating distinct taxonomic groups.

## References

Borgmann U. Systematic analysis of aqueous ion requirements of Hyalella azteca: A standard artificial medium including the essential bromide ion*. Archives of Environmental Contamination and Toxicology*. 1996; 30, 356–363.

Kuhl A, Lorenzen H. Chapter 10 Handling and Culturing of Chlorella. *In D. M. Prescott (Hrsg.), Methods in Cell Biology (Bd. 1, S. 159–187). Academic Press.* 1964 https://doi.org/10.1016/S0091-679X(08)62092-0

Liu C, Cui Y, Li X et al. microeco: An R package for data mining in microbial community ecology. *FEMS Microbiology Ecology.* 2021; *97*(2), fiaa255. https://doi.org/10.1093/femsec/fiaa255

Rybicki M, Jungmann D. Direct and indirect effects of pesticides on a benthic grazer during its life cycle. *Environmental Sciences Europe.* 2018; 30(1), 35. https://doi.org/10.1186/s12302-018-0165-x

Rybicki M, Winkelmann C, Hellmann C et al. Herbicide indirectly reduces physiological condition of a benthic grazer*. Aquatic Biology.* 2021; 17(2), 153–166. https://doi.org/10.3354/ab00472
